# Longitudinal study of patients with chronic Chagas cardiomyopathy in Brazil (SaMi-Trop project): a cohort profile

**DOI:** 10.1136/bmjopen-2016-011181

**Published:** 2016-05-04

**Authors:** Clareci Silva Cardoso, Ester Cerdeira Sabino, Claudia Di Lorenzo Oliveira, Lea Campos de Oliveira, Ariela Mota Ferreira, Edécio Cunha-Neto, Ana Luiza Bierrenbach, João Eduardo Ferreira, Desirée Sant'Ana Haikal, Arthur L Reingold, Antonio Luiz P Ribeiro

**Affiliations:** 1 Federal University of São João del-Rei, Brazil; 2 University of California, Berkeley, California, USA; 3 Department of Infectious Diseases, School of Medicine and Institute of Tropical Medicine, University of São Paulo, São Paulo, Brazil; 4 Laboratory of Medicine Laboratorial (LIM03), General Hospital, School of Medicine, University of São Paulo, São Paulo, Brazil; 5 Health Science Programme, State University of Montes Claros, Montes Claros, Minas Gerais, Brazil; 6 Laboratory of Immunology, Heart Institute (InCor), School of Medicine, University of São Paulo, São Paulo, Brazil; 7 Division of Clinical Immunology and Allergy, School of Medicine, University of São Paulo, São Paulo, Brazil; 8 Institute for Investigation in Immunology, iii-INCT, São Paulo, Brazil; 9 Institute of Mathematics and Statistics (IME), University of São Paulo, São Paulo, Brazil; 10 Department of Internal Medicine, Universidade Federal de Minas Gerais, Belo Horizonte, Brazil

**Keywords:** Chagas disease, Cohort Studies, Biomarkers, CHEMICAL PATHOLOGY

## Abstract

**Purpose:**

We have established a prospective cohort of 1959 patients with chronic Chagas cardiomyopathy to evaluate if a clinical prediction rule based on ECG, brain natriuretic peptide (BNP) levels, and other biomarkers can be useful in clinical practice. This paper outlines the study and baseline characteristics of the participants.

**Participants:**

The study is being conducted in 21 municipalities of the northern part of Minas Gerais State in Brazil, and includes a follow-up of 2 years. The baseline evaluation included collection of sociodemographic information, social determinants of health, health-related behaviours, comorbidities, medicines in use, history of previous treatment for Chagas disease, functional class, quality of life, blood sample collection, and ECG. Patients were mostly female, aged 50–74 years, with low family income and educational level, with known Chagas disease for >10 years; 46% presented with functional class >II. Previous use of benznidazole was reported by 25.2% and permanent use of pacemaker by 6.2%. Almost half of the patients presented with high blood cholesterol and hypertension, and one-third of them had diabetes mellitus. N-terminal of the prohormone BNP (NT-ProBNP) level was >300 pg/mL in 30% of the sample.

**Findings to date:**

Clinical and laboratory markers predictive of severe and progressive Chagas disease were identified as high NT-ProBNP levels, as well as symptoms of advanced heart failure. These results confirm the important residual morbidity of Chagas disease in the remote areas, thus supporting political decisions that should prioritise in addition to epidemiological surveillance the medical treatment of chronic Chagas cardiomyopathy in the coming years. The São Paulo-Minas Gerais Tropical Medicine Research Center (SaMi-Trop) represents a major challenge for focused research in neglected diseases, with knowledge that can be applied in primary healthcare.

**Future plans:**

We will continue following this patients’ cohort to provide relevant information about the development and progression of Chagas disease in remotes areas, with social and economic inequalities.

**Trial registration number:**

NCT02646943; Pre-results.

**Strengths and limitations of this study**

- In this large multicentre cohort of patients with Chagas cardiomyopathy previous use of benznidazole was reported by one quarter of the patients.
- Clinical and laboratory markers predictive of severe and progressive Chagas disease (ChD) were identified in the São Paulo-Minas Gerais Tropical Medicine Research Center (SaMi-Trop) cohort, as high N-terminal of the prohormone BNP (NT-ProBNP) levels, as well as symptoms of advanced heart failure.
- Results presented in this paper confirm the important residual morbidity of ChD in remote areas, thus supporting political decisions that should prioritise in addition to epidemiological surveillance the medical treatment of CCC in the coming years.
- The SaMi-Trop cohort represents a major challenge for focused research in neglected diseases, with knowledge that can be applied in primary health care.
- One weakness is the lack of baseline echocardiograms, which could help in the clinical stratification of patients. However, this information is being collected in the second follow-up visit.

## Introduction

Chagas disease, which is caused by the protozoan parasite *Trypanosoma cruzi*, remains one of the most neglected diseases in the world, with 8–10 million infected people. The most important consequence of Chagas disease is chronic Chagas cardiomyopathy, which occurs in 20–40% of infected persons,^1–4^ with an incidence rate of 1.85% person-year.^4^

Chronic Chagas cardiomyopathy comprises a wide range of manifestations, including heart failure, arrhythmias, heart blocks, sudden death, thromboembolism, and stroke.^5^ Clinical presentation typically varies widely according to the degree of myocardial damage, and most patients present a mild form of heart disease frequently characterised only by the presence of asymptomatic abnormalities on the ECG or in other complimentary examinations.^6^ The Brazilian Consensus of Chagas disease defines Chagas cardiomyopathy as the presence of typical ECG abnormalities in patients with a positive serological test for *T. cruzi* infection.^7^ When heart failure and/or severe arrhythmias manifest, the prognosis is ominous, with high and premature mortality rates in adult male patients,^8^ as well as in the elderly.^9^ Indeed, when compared with patients with idiopathic cardiomyopathy, patients with chronic Chagas cardiomyopathy have poorer survival, irrespective of other clinical and echocardiographic parameters.^10^

Chronic Chagas cardiomyopathy is a potentially lethal condition, but the severity of the disease varies widely and accurate stratification of the risk of disease progression and death remains an unsolved challenge.^5^ Risk scores have been developed,^11–13^ including a validated one.^11^ However, current risk scores rely on the availability of several diagnostic tests, including Holter monitoring, stress testing, echocardiographic examination and chest X-ray,^11^ or special examinations such as signal averaged ECG.^12^ ^13^ These methods are not readily available in the rural endemic areas and have a limited role in risk stratification in the primary care setting. Indeed, a simple, low-cost and easy-to-use prognostic model suitable for the primary care setting is lacking. Although some promising studies show the potential value of some new biomarkers,^14^ ^15^ the lack of validated and easily available biomarkers for active infection or clinical end points are a problem for assessing the performance of new drugs or therapeutic interventions. In addition, given the lack of a health service structure, mainly in remote areas, along with the low levels of awareness among healthcare providers, cases of chronic Chagas cardiomyopathy are under-recognised and undertreated.

Seeking to contribute to the knowledge of Chagas disease, a large cohort of patients with chronic Chagas cardiomyopathy was established in Minas Gerais State (Brazil). This cohort aims to develop a prognostic algorithm—based on simple ECG measurements in conjunction with clinical information and brain natriuretic peptide (BNP) levels—that would be used to predict the risk of disease progression and death in patients with chronic Chagas cardiomyopathy and be useful in the clinical management of such patients. This paper outlines the study and baseline characteristics of the cohort participants.

## Cohort description

**Figure 1:**
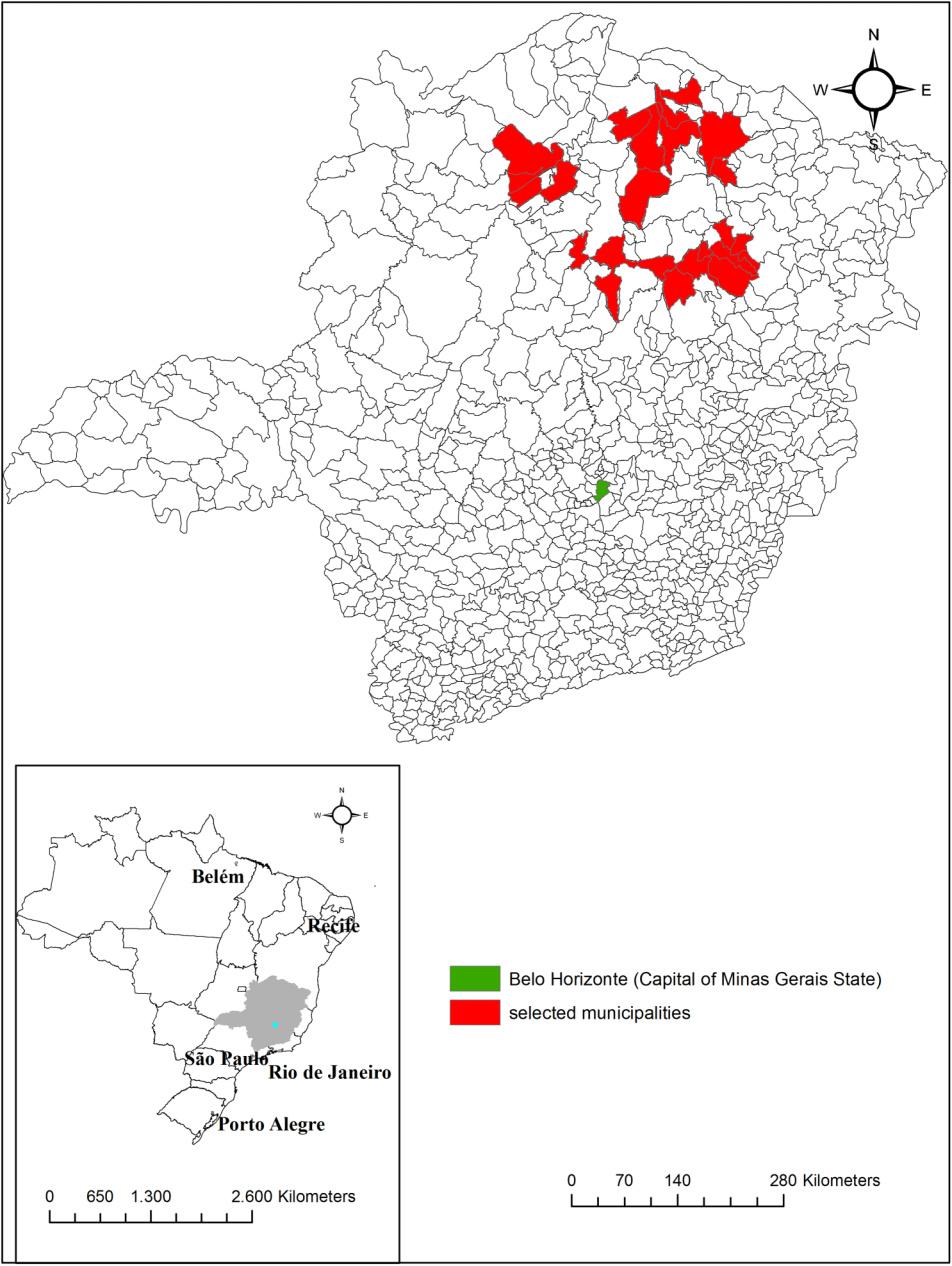
Geographical location of the 21 municipalities included in the São Paulo-Minas Gerais Tropical Medicine Research Center (SaMi-Trop) project. Minas Gerais, Brazil.

The São Paulo-Minas Gerais Tropical Medicine Research Center (SaMi-Trop) consists of a network of collaborating scientists in the States of Minas Gerais and São Paulo which has been established for the purpose of developing and conducting research projects on Chagas disease. The SaMi-Trop project is a prospective cohort study with at least 2 years of follow-up, including one visit at baseline and another at 24 months. The cohort of patients with chronic Chagas cardiomyopathy was established by using patients under the care of the Telehealth Network of Minas Gerais, a programme designed to support primary care in Minas Gerais State, Brazil.^16^ In this programme, all patients' ECG and clinical data are sent to a central reading unit centre that also collects clinical data such as the history of Chagas disease. Using this database, we selected 21 municipalities within a limited region in the northern part of the State of Minas Gerais where the prevalence of patients with chronic Chagas cardiomyopathy was expected to be high (figure 1).

Eligible patients were selected based on the ECG results performed in 2011–2012 by the Telehealth Network, which from now on will be called index ECG. Only patients who fulfilled all of the following inclusion criteria were selected: (1) self-reported Chagas disease; (2) presence of the following abnormalities on the index ECG:^17^ possible old myocardial infarction (major Q wave abnormalities or minor Q waves abnormalities with ST segment or T wave abnormalities), complete intraventricular block (right, left or unspecified), frequent supraventricular or ventricular premature beats, major isolated ST segment or T wave abnormalities, atrial fibrillation or flutter or supraventricular tachycardia or other major arrhythmias, major atrioventricular conduction abnormalities or pacemaker use, or major QT prolongation (QT index >115%), left or right ventricular hypertrophy and (3) aged 19 years or more. The exclusion criteria included pregnancy or breast feeding, and any life-threatening disease with an ominous prognosis that suggested a life expectancy of <2 years.

The simple size was calculated considering the minimal number of events per variable acceptable in a proportional hazards regression analysis of 10 events per variable.^18^ As the prediction model has to be developed and validated, and the whole sample will be divided into two, the number of events should be 200. For a 2-year follow-up period and annual mortality rate of 5% in chronic Chagas cardiomyopathy (10% in 2 years), the calculated sample size was 2000 participants.

All eligible participants were tested for the presence of anti-*T. cruzi* antibodies using chemiluminescent microparticle immunoassay. Negative results were confirmed by two other enzyme immunoassay (EIA) presenting different antigens. The final cohort consists of patients confirmed to be seropositive. The patients’ distribution by municipalities and the distance from the reference centre in health is presented in table 1.

**Table 1:** Distribution of patients including in São Paulo-Minas Gerais Tropical Medicine Research Center (SaMi-Trop) cohort according to the municipality and distance to the reference centre, Montes Claros (n=1959)

Municipalities	Number	Per cent	Distance (km)
São Francisco	325	16.6	163
Carbonita	202	10.3	203
Minas Novas	164	8.4	289
Janaúba	166	8.5	134
Turmalina	131	6.7	264
Bocaiúva	128	6.5	47
Chapada do Norte	122	6.2	295
Berilo	113	5.8	333
Porteirinha	71	3.6	170
Brasília de Minas	71	3.6	105
Fruta de Leite	68	3.5	186
Claro dos Poções	62	3.2	79.5
Verdelândia	69	3.5	173
Pai Pedro	56	2.9	185
Ubaí	54	2.8	153
Leme do Prado	42	2.1	273
Francisco Sá	38	1.9	52
Rio Pardo de Minas	28	1.4	276
Jenipapo de Minas	19	1.0	369
Francisco Badaró	16	0.8	347
Monte Azul	14	0.7	244
Total	1959	100.0	–

The cohort will be followed for 2 years, until primary outcome or loss to follow-up. The primary outcome is death and the secondary outcomes are changes in the ECG pattern and hospitalisation due to cardiovascular complications. Ascertainment of the occurrence of deaths will be done using the National Mortality Information System (SIM) from the Ministry of Health.

Table 2 summarises the types of data collected at baseline and of those that will be collected at the 2-year follow-up visit. All eligible participants were recruited by the family health programme team. The baseline visit was performed at public health primary care units by previously trained staff. The patients were interviewed using a standardised questionnaire, and had a blood sample collected and an ECG evaluation. The data were collected electronically and sent to the data centre at the University of São Paulo via a web-based system.

**Table 2:** Measurements obtained at different phases of the SaMi-Trop study

Phase	Measurements
Baseline: 2013–2014	Questionnaires with sociodemographic information, social determinants of health, health-related behaviours (smoking, alcohol consumption and physical activity), self-reported comorbidities, medication use, history of previous treatment for Chagas disease, signs and symptoms, functional class (Cardiovascular Functional Class Scale)^19^ and quality of life (WHO-QOL-BREF)^20^ ECG Blood collection: immunoassays, PCR for *T. cruzi*, NT-ProBNP
Follow-up: 2015–2016	Questionnaires with sociodemographic information, social determinants of health, health-related behaviours, self-reported comorbidities, medication use, signs and symptoms since baseline. Functional class and quality of life Vital status, history of health service utilisation, including hospital admission^a^, health literacy (Short Assessment of Health Literacy)^21^ ECG Echocardiogram Blood collection: NT-ProBNP

NT-ProBNP, N-terminal of the prohormone brain natriuretic peptide; SaMi-Trop, São Paulo-Minas Gerais Tropical Medicine Research Center; *T*. *cruzi*, *Trypanosoma cruzi*; WHO-QOL-BREF, WHO Quality of Life-BREF.

A resting 12-lead ECG was recorded using an ECG PC machine (TEB, São Paulo, Brazil). The ECG recordings were sent electronically to the Telehealth system and read by a trained cardiologist; the written report was subsequently returned to the patient’s physician. For research purposes, ECGs were also automatically analysed using the University of Glasgow ECG analysis programme (release 28.5, issued on January 2014) and reviewed by trained cardiologists to ensure quality control. ECGs will be classified using the Minnesota Code criteria using variables derived from the median complex of the Glasgow University software measurement matrix.^22^

Blood was collected into serum-separating tubes, and allowed to clot at room temperature for 30 min. The serum was centrifuged at 1300 g for 10 min at room temperature. This was then subjected to storage at −20°C and later shipped with dry ice to the central laboratory in São Paulo.

Brazilian Mortality Information (SIM) data will be used to ascertain patients’ vital status after the follow-up period as well as the underlying causes of death, which are coded under the International Classification of Disease, 10th Revision (ICD-10).

In this paper, a descriptive analysis of the baseline characteristics of the cohort participants was done using frequency and percentage distribution. SPSS V.19 (SPSS Inc, IBM, Armonk, New York, USA) and ArcView, V.10.1 (Environmental Systems Research Institute Inc, http://www.esri.com/software/arcview/) were used.

This cohort study is a component of a larger study to evaluate biomarkers of Chagas disease sponsored by a grant from National Institute of Allergy and Infectious Diseases (NIAID)/National Institutes of Health (NIH) Neglected Tropical Disease Centre.

## Characteristics of the study population

**Figure 2:**
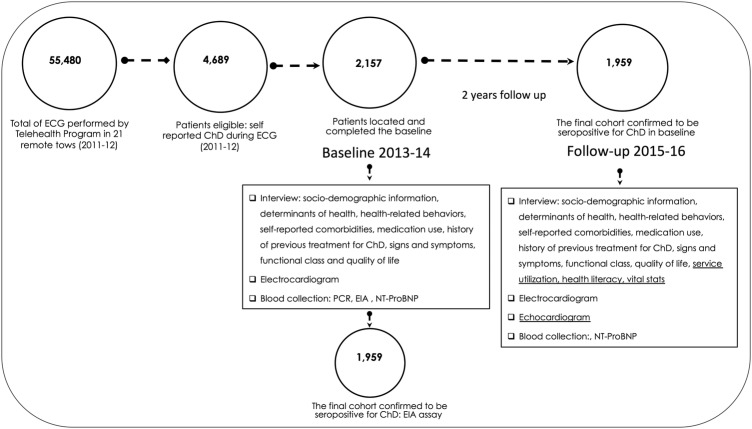
The SaMi-Trop project diagram. ChD, Chagas disease; EIA, enzyme immunoassay; NT-ProBNP, N-terminal of the prohormone brain natriuretic peptide; SaMi-Trop, São Paulo-Minas Gerais Tropical Medicine Research Center.

Of the 55 480 ECGs performed in the 21 selected municipalities from 2011 to 2012, a total of 4689 patients were eligible for the study, and 2157 were located and completed the baseline assessment in 2013–2014. In comparison to the eligible group, the participants had a higher percentage of women (67.1% vs 59.9%, p<0.01) and were younger (59.5 vs 60.7 years, p<0.01). The final cohort consists of 1959 (90.8%) participants confirmed to be seropositive for Chagas disease (figure 2).

Table 3 shows the main sociodemographic characteristics, socioeconomic categories and self-perception of health of study participants at baseline. Most patients were female (67.5%), aged between 50 and 74 years (62.6%), sharing the same household with two other people or less (56.6%), and had a family monthly income of US$327. The educational level was very low with 38.7% having had between 1 and 4 years of school, and 34.4% never having attended school. Cohort members self-reported their health status as average (57.7%) or good (25.8%).

**Table 3:** Sociodemographic characteristics of SaMi-Trop cohort members (n=1959)

Variables	N	Per cent
Sex		
Female	1.323	67.5
Male	636	32.5
Age		
<50 years	499	25.6
50–74 years	1.223	62.6
>74 years	231	11.8
Number of household members		
1–3	1.106	56.6
4–6	709	36.3
7–17	138	7.1
Family monthly income data		
>US$327	1.037	53.1
≤US$327	916	46.9
Skin colour		
Mixed	1.144	58.6
White	426	21.8
Black	348	17.8
Others	32	1.8
Years of school		
1–4 year	862	44.2
Illiterate	670	34.4
5–8 years	320	16.4
Other	98	5.0
Marital status		
Married or living with partner	1.238	63.4
Widower	449	23.0
Single	176	9.0
Divorced	90	4.6
Self-perception of health		
Very good	57	2.9
Good	499	25.8
Average	1.116	57.7
Bad or very bad	264	13.6

Small differences in total N for each variable are due to missing values.

Others in Skin colours include Asians (27) and Native Americans (5).

Others in Educational level included: elementary school (81) and graduate school (17).

Dollar quotation from July 2013.

The majority of the patients self-reported that they have had Chagas disease for over 10 years, and that they had at least one family member with a history of Chagas disease. Based on the New York Heart Association (NYHA) Functional Classification, 45.9% of the patients were classified as level II or more (ie, have symptoms of heart failure). Among Chagas disease patients in the cohort, 6.2% reported permanent use of a pacemaker. Previous treatment for Chagas disease was reported by 51.6% of Chagas disease patients (25.2%), including 492 who reported previous treatment with benznidazole. The N-terminal of the prohormone BNP (NT-ProBNP) level was >300 pg/mL in 30% of the sample (table 4).

**Table 4:** Distribution of patients according to self-reported Chagas disease, cardiovascular functional class and NT-ProBNP results in the SaMi-Trop study

Variables	Valid N	N	Per cent
Chagas disease self-reported	1.955		
Yes		1870	95.6
No		64	3.3
No response		21	1.1
Duration of Chagas disease (years)	1.896		
>10		1179	62.2
1–10		695	36.6
<1		22	1.2
Chagas disease in another family member	1.947		
Yes		1384	71.1
No		384	19.7
Do not know		179	9.2
Previous treatment for Chagas disease	1.953		
Yes		1008	51.6
No		873	44.7
Do not know		72	3.7
Previous use of benznidazole medicine	1.955		
No		1320	67.5
Yes		492	25.2
Do not know		143	7.3
NYHA Functional Classification	1.931		
I		1059	54.8
II or more		872	45.2
NT-ProBNP level, pg/mL	1.955		
<300		1368	70.2
≥300		581	29.8

NT-ProBNP, N-terminal of the prohormone brain natriuretic peptide; NYHA, New York Heart Association.

As seen in table 5, the prevalence of one or more self-reported comorbid conditions at baseline was high, including high serum cholesterol (40.1%), hypertension (36.0%), diabetes mellitus (10.1%), thyroid disorder (8.1%), and kidney disease (7.3%). Leishmaniasis was reported by 22 patients (1.2%). Only 22.3% of patients reported having performed any physical activity during the prior week, 16.2% reported having drunk alcohol in the previous month, and 7.3% reported that they were current smokers. In terms of medications, 36.4% of patients reported the current use of one or two medicines, while 30.1% reported no current use of any medication. The most common medicines being used were diuretics (49.1%), ACE (28.6%), angiotensin receptor blockers (ARBs) (28.4%), aspirin (26.2%), and amiodarone (22%).

**Table 5:** Prevalence of comorbid conditions, selected behavioural characteristics, medications used, signs and symptoms, and self-reported health in the SaMi-Trop cohort.

Variables	Valid N	N	Per cent
Comorbid conditions	1.959		
High serum cholesterol		785	40.1
Hypertension		706	36.0
Diabetes mellitus		198	10.1
Thyroid disorder		159	8.1
Kidney disease		143	7.3
Leishmaniosis		22	1.2
Behavioural characteristics	1.945		
Physical activity last week (minimal 30 min)		434	22.3
Alcohol last month		318	16.2
Current smoking		143	7.3
Number of medicine in use	1.959		
0		589	30.1
1–2		714	36.4
3–4		538	27.5
≥5		118	6.0
Medicine in use (yes)	1.940		
Diuretics		951	49.1
ACE		553	28.6
ARBs		550	28.4
Aspirin		507	26.2
Amiodarone		429	22.0
Carvedilol		380	19.6
Digoxin		140	7.2
β-blockers		140	7.2
Vasodilators		84	4.3
Warfarin		11	0.6
Signs and symptoms or self-reported conditions (yes)	1.924		
Heartbeat racing or beating abnormally		1.222	63.5
Prolonged faintness or dizziness		1.203	62.5
Problems on ECG		1.180	61.3
Heart palpitations		1.174	61.0
Short of breath during physical exercises		1.143	59.4
Heartbeat racing at rest		1.015	52.8
Heartbeat not regular		902	46.9
Difficulty breathing when lying down		752	39.1
Unable to climb two flights of stairs		749	38.9
Awake during the night unable to breath		683	35.5
Trouble swallowing		599	31.1
Swelling or puffiness of the feet in the morning		502	26.1
No bowel movement for three or more days		478	24.8
Fainting or loss of consciousness		429	22.0
Visible neck veins when standing up or sitting		409	21.3
Pain when swallowing food		342	17.8
Pacemaker		110	6.2
Megaesophagus		117	6.1

ARBs, angiotensin receptor blockers; SaMi-Trop, São Paulo-Minas Gerais Tropical Medicine Research Center.

Considering the fact that almost half of the patients were in functional class II or more, with 30% with BNP levels higher than 300 units/L, there is an overall low usage of the recommended drugs for heart failure, particularly of β-blockers. This may explain at least in part the high frequency of cardiac symptoms reported: 63.5% had racing heartbeat, 62.5% had prolonged fainting spells or dizziness, 61.3% had an abnormal ECG, 61.0% had heart palpitations, and 59.4% had shortness of breath at exercises. The relative high frequency of amiodarone use may be related to the high frequency of cardiac arrhythmias in chronic Chagas cardiomyopathy, as well as the established practice, in Brazil, to prescribe amiodarone to prevent sudden death.

## Findings to date

Clinical and laboratory markers predictive of severe and progressive Chagas disease were identified in SaMi-Trop cohort, as high NT-ProBNP levels, as well as symptoms of advanced heart failure. The NT-ProBNP level was >300 pg/mL in 30% of the sample. High circulating levels of natriuretic peptides are related to the presence of left ventricular dysfunction^23^ and a higher risk of death.^24^

Among Chagas disease patients in the cohort, 6.2% reported permanent use of a pacemaker. This percentage is far below the 14.0% found in another recently published Brazilian cohort study of patients with Chagas disease.^25^ However, Chagas disease is still a major cause of use of pacemakers and defibrillators in Brazil, surpassing the indications due to coronary artery disease in some regions. The literature too point out the underuse of this device in Brazil when compared with other countries,^26^ which is unfortunately what we expected to find in the remote regions represented in the current study.

In this large multicentre cohort, previous use of benznidazole was reported by one-fourth of the patients. It is well known that the persistence of *T*. *cruzi* is directly related in the pathology of the chronic phase, but it remains to be proved that parasite load reduction by trypanocidal treatment leads to concomitant attenuation of cardiomyopathy.^2^ ^25^ In the recently released BENEFIT trial,^25^ that included Brazilian patients, treatment with benznidazole did not significantly reduce cardiac clinical deterioration in the 5 years of follow-up in Chagas cardiomyopathy.

Although *T. cruzi* infection does not have a sexual predilection,^27^ studies show higher prevalence among women.^28^ ^29^ This difference may be related to frequent use of health services by women, even after controlling for restrictions in routine activities due to health reasons^30^ as well as greater availability to participate in scientific studies, especially those with a longitudinal component such as in this investigation.

Results presented in this paper confirm the important residual morbidity of Chagas disease in the remote areas, thus supporting political decisions that should prioritise in addition to conducting epidemiological surveillance of the medical treatment for chronic Chagas cardiomyopathy in the coming years. The SaMi-Trop cohort represents a major challenge for focused research in neglected diseases, with knowledge that can be applied in primary healthcare. The study has the potential to provide relevant information about the development and progression of Chagas disease in remote areas with social and economic inequalities.

## Strengths and limitations

The SaMi-Trop is one of the largest multicentre cohort study of Chagas disease conducted in the world. It has the potential of identifying biomarkers that will be used to predict the risk of disease progression and death, as well as permit comparative analysis with other similar cohorts. Most studies that evaluated biomarkers in Chagas disease had a cross-sectional design. The large number of patients included in this investigation is outstanding, especially in a rural and dispersed area. Our preliminary results confirmed the important residual morbidity of Chagas disease in such remote areas and found that these patients are currently being undertreated. We hope that our findings will guide political decisions aiming at enhancing access to healthcare of Chagas disease patients in the coming years.

Second, the SaMi-Trop cohort represents a major challenge for focused research in neglected diseases, with knowledge that can be applied in primary healthcare. The study has the potential to provide relevant information about the development and progression of Chagas disease in remote areas with social and economic inequalities. As pointed out by Maguire,^31^ there is an urgent need for a new strategy for Chagas disease treatment and studies for evaluation of results because the infected patients are ageing and have only a few years left to live.

One weakness of the study is that no data on weight and height in the health services indicators were included at the baseline. Another important limitation is the lack of baseline echocardiograms, which could help in the clinical stratification of patients. All this information is being collected in the second follow-up visit. As the focus was to find biomarkers related to the cardiac outcome and given the budgetary limitations, no indeterminate form or negative controls were included in this cohort; this will preclude the study of the early biomarkers of disease progression. However, Chagas disease is mostly a disease of adults and older age groups in countries where the vectorial transmission was interrupted and this cohort provides a unique opportunity for recognising predictors of higher risk using simple biomarkers in a community sample of patients with Chagas cardiomyopathy.

## Collaborations

Collaborations in data analysis will be welcome through specific research proposals sent to individual SaMi-Trop investigators. Exchange of doctoral or postdoctoral fellows is very welcome.

The data set will be open access for two years at the end of the data collection process (August 2018). In the meantime, applications to use the data should be made by contacting the researchers of the SaMi-Trop cohort and filling in the application form. The questionnaires and interviewer guides of the baseline will also be available in electronic formats at http://www.ufsj.edu.br/tecnologiasemsaude_pesquisa/projetos.php.
